# Autobiographical Memory and Social Identity in Autism: Preliminary Results of Social Positioning and Cognitive Intervention

**DOI:** 10.3389/fpsyg.2021.641765

**Published:** 2021-03-17

**Authors:** Prany Wantzen, Amélie Boursette, Elodie Zante, Jeanne Mioche, Francis Eustache, Fabian Guénolé, Jean-Marc Baleyte, Bérengère Guillery-Girard

**Affiliations:** ^1^Normandie Univ, UNICAEN, PSL Université Paris, EPHE, INSERM, U1077, CHU de Caen, GIP Cyceron, Neuropsychologie et Imagerie de la Mémoire Humaine, Caen, France; ^2^Service de Psychiatrie de l'enfant et de l'adolescent, CHU de Caen, Caen, France; ^3^Service de Psychiatrie de l'enfant et de l'adolescent, Créteil University Hospital, Créteil, France

**Keywords:** autism spectrum disorders, autobiographical memory, self, therapeutic interventions, social positioning, social identity, self-categorization

## Abstract

Autobiographical memory (AM) is closely linked to the self-concept, and fulfills directive, identity, social, and adaptive functions. Individuals with autism spectrum disorder (ASD) are now known to have atypical AM, which may be closely associated with social communication difficulties. This may result in qualitatively different autobiographical narratives, notably regarding social identity. In the present study, we sought to investigate this concept and develop a cognitive intervention targeting individuals with ASD. First, 13 adolescents with ASD and 13 typically developing adolescents underwent an AM interview featuring an original coding system designed to analyze the social self. We observed that the narratives produced by the ASD group focused more on the family than on extended social spheres, compared with those of the comparison group. Moreover, participants with ASD did not include themselves in the social groups they mentioned, and produced more references to others, compared with typically developing participants. Second, we designed a cognitive intervention program consisting of individual and group sessions that targeted AM. We conducted a pilot study among three adolescents with ASD aged 12, 16, and 17 years. Preliminary results showed that the program increased extra-family narrative references by the two youngest adolescents, who produced more social integration markers. Our study of autobiographical narratives yielded interesting findings about social positioning in ASD and showed how AM can be targeted in rehabilitation programs as a vector of social interaction.

## General Introduction

Autism spectrum disorder (ASD) is a neurodevelopmental disorder characterized by restricted and repetitive behaviors, together with social communication difficulties (American Psychiatric Association, 2013). These difficulties include theory of mind (ToM) impairment that interfere with developing, maintaining and understanding relationships. Individuals with ASD display atypical behavioral adjustment to social contexts, as well as problems making friends and initiating a conversation or keeping one going. All these abilities significantly impact their ability to construct a social identity and define their personal identity relative to social groups (social identity theory; Tajfel and Turner, 1979). In the typically developing (TD) population, social identities are forged through validating and consensual social interactions. The social communication and adjustment difficulties that characterize individuals with ASD may therefore interfere with their development of social identities through social interaction. They may hinder their sharing of experiences with members of the different groups to which they belong, their elaboration of psychosocial connections to these groups, and the degree to which individuals with ASD feel included as full group members. Social identity has attracted very little research interest, even though it is related to mental health and may be a protective mechanism, as recently demonstrated by Cooper et al. (2017). Autobiographical memory (AM) may provide a means of investigating social identity markers that are closely related to ToM and social skills (Perner and Ruffman, 1995; Welch-Ross, 1997). In particular, analyzing narratives production may yield information on social positioning within a specific group, and within and outside the family, with reference to social identities.

AM includes long-term memory of general personal knowledge or facts (i.e., semantic AM) and specific events related to individuals' own lives (i.e., episodic AM). This episodic component allows individuals to remember past experiences (i.e., episodic autobiographical memories) and also to imagine possible future experiences (i.e., episodic future thinking; Tulving, 1985). Both episodic memories and future thinking involve autonoetic consciousness, namely the ability to project states of self into time. Autobiographical information about the past and the projection into the future allows individuals to retain a sense of being a coherent person throughout their lifetime and to update their self-identity while maintaining continuity (Conway, 2005). Thence, maintaining *self* or *identity* is one of AM's three main functions, along with directive and social processes (Bluck and Alea, 2002).

The *directive* function consists in using past experiences to guide present and future thoughts, actions, and behaviors (Baddeley, 1988; Bluck et al., 2005; Williams et al., 2008), and to solve problems. It is also involved in ToM, that is, the understanding of others' inner world, and thence the prediction of their future behavior (Robinson and Swanson, 1990). The *social* function is the third essential role of AM (Neisser, 1988). AM makes it easier for individuals to initiate, develop, and maintain social interactions and relations through conversation (Pillemer, 1998; Nelson, 2003; Bluck et al., 2005). Sharing personal experience and knowledge facilitates understanding and empathy in social interactions (Alea and Bluck, 2003). Past experiences provide the information needed to better attribute and understand the emotions and feelings of others. Through self-disclosure processes, AM allows very close relationships to be developed with people who share memories or provide the listener with information about oneself if someone was not present at the event (Laurenceau et al., 1998; Alea and Bluck, 2007). AM can be interpreted as an individual memory, but may extend to collective memory, in the case of an event that people experienced together. When this collective memory is shared with family, friends or acquaintances, it can create or maintain a social network. By the same token, a social network can calibrate the contents of individual memory (Halbwachs, 1925).

Among the few studies to have explored self-related AM in ASD, several have focused on personal autobiographical knowledge (i.e., physical, moral, sociocultural, and psychological). This semantic component of AM seems to be preserved in adults with ASD (Goddard et al., 2007; Tanweer et al., 2010; Crane et al., 2012; Zamoscik et al., 2016), but not in children with ASD (Bruck et al., 2007; Goddard et al., 2014). More specifically, semantic knowledge related to the concept of the psychological self is impaired, whereas that related to the physical self is preserved (Bruck et al., 2007; Bon et al., 2012; Brown et al., 2012; Goddard et al., 2014; Robinson et al., 2016). This pattern may be due to delayed development of identity (Lind, 2010). In their longitudinal study, Bon et al. (2012) observed a gradual forgetting of personal knowledge, suggesting a semantization or consolidation impairment that may stem from diminished rehearsal associated with impaired social interactions. A study revealed that individuals with high autistic traits had a less clear self-concept, related to a reduced ability to make meaning of important past life events (Berna et al., 2016).

The episodic component of AM (i.e., focused on personal events) is impaired in ASD, with reduced retrieval, specificity, elaboration and episodic coherence, and a decrease in details, particularly narrative, perceptual, emotional and cognitive ones (Bruck et al., 2007; Goldman, 2008; Brown et al., 2012; Lind et al., 2014; Anger et al., 2019), affecting both past memories and projections into the future (Crane et al., 2010; Lind and Bowler, 2010; Terrett et al., 2013; Marini et al., 2019). The lack of contextual details and access to information also contributes to the aberrant formation of a sense of identity. These impairments are linked to a decrease in self-experience, use of the self as an efficient organization system memory, and labeling of information as being related to the self (Millward et al., 2000; Goddard et al., 2007; Crane et al., 2010; Coutelle et al., 2020). Studies in ASD report poorer mentalization and social functioning abilities, especially among individuals with more severe autistic traits, in line with difficulty recalling self-relevant events (Lombardo et al., 2007; Henderson et al., 2009). Moreover, children with ASD fail to incorporate autobiographical details in their narratives to promote shared memories in a social context (Goldman, 2008). Individuals with ASD fail to grasp the value of using memory as an object of social interaction. However, no study has explored AM in ASD to investigate social positioning in different groups. Moreover, AM has never been used as a vector of social interaction within a care program intended to improve social abilities.

### Current Study

In this context, we conducted two studies of social identity in ASD. Study 1 investigated social identity through the autobiographical narratives of adolescents with ASD without intellectual disabilities (10-19 years), compared with those of TD adolescents. Adolescence is a crucial period for social identity. We predicted that discourse would be more self-centered, with a less adequate narrative structure, less social inclusion, and more self-references, in the ASD group than in the TD group. Study 2 reinvestigated our results in a novel cognitive remediation program targeting AM in three adolescents with ASD without intellectual disabilities, aged 12, 16, and 17 years. We predicted that the remediation would positively impact their social autobiographical narratives. Individual sessions offered personalized follow-up after an initial assessment, and group sessions targeted shared memory. We predicted that the adolescents with ASD would have a better social identity process at the end of the program, extending beyond the family to a wider circle.

## Study 1: Autobiographical Memory and Social Identity

### Introduction

The self can be understood through the groups with which individuals identify themselves (Tajfel and Turner, 1979). The social self approach is based on social identity theory: intra- and intergroup behaviors vary according to how the members of one group perceive themselves in relation to other groups. It also refers to self-categorization: how individuals perceive themselves and others as a group and the consequences of these perceptions (Hornsey, 2008). The self also has an interpersonal dimension, modulated by interactions built in part by contrast with others and others shape identity (Baumeister, 2011).

Turner and Reynolds (2011) postulated that there are three abstraction levels in self-categorization theory. The lowest level corresponds to the personal self (i.e., intrapersonal level; individual and personal characteristics with no reference to others). The next level concerns the social self, at an *interpersonal* (i.e., “I am a unique individual compared with others”) or *intergroup* (i.e., “I define myself as the member of one group relative to another group”) level. The highest level of abstraction corresponds to the notion of “we are humans *vs*. animals or other non-humans.” The social self level can be divided into three categories: egocentric, intracentric, and allocentric. *Egocentric* is when the speaker is present but not included in a social group (*self-reference*; e.g., “I went to the beach with my friend”). *Intracentric* is when the speaker is included in a social group (*inclusive reference*; e.g., “We went to the beach with my friend”). *Allocentric* is when the speaker is not present and not included in a social group (*reference to others*; e.g., “My friend went to the beach”).

Children with ASD produced fewer self-statements to define themselves, using more abstract, less specific, and less social terms than their TD peers (Lee and Hobson, 1998; Tanweer et al., 2010; Jackson et al., 2012). Coutelle et al. (2020) showed that adults with ASD exhibit reduced clarity of self-concept, compared with TD adults. They experienced their AM with a lower social binding function and more for the purpose of self-understanding. Adults with ASD reported difficulty attributing internal mental states both to themselves (i.e., introspection) and to others (i.e., ToM; Perner et al., 1989; Williams and Happé, 2009). Robinson et al. (2016) also described introspection and mentalizing impairments in a sample of children with ASD, who displayed less knowledge about others' internal mental states than TD participants did. Individuals with ASD assumed that someone close to them has a better perception of their own behavior than they do themselves (Robinson et al., 2016).

In a meta-analysis about narratives produced by high-functioning children with ASD, Baixauli et al. (2016) reported poorer narrative performances for children with ASD than for TD, with ambiguous use of pronouns. As we saw earlier, conversational narratives—particularly AM narratives—play a crucial role in the sharing of experiences and the development of social relationships (Klitzing et al., 2007). AM is linked to social interaction, enabling individuals to establish and maintain social ties (Bluck et al., 2005) and facilitating social inclusion. Moreover, how we perceive ourselves in memory depends in part on the interactions we have with others (Wilbers et al., 2012). Finally, given the bidirectional relationship between the self and AM, it is speculated that these difficulties subtend the reduced impact of AM's social function in ASD and contribute to social impairment. We therefore investigated how the social self is reflected in AM narration and how individuals with ASD categorize themselves within the different social spheres.

### Material and Methods

#### Participants

We recruited 13 adolescents (11 males) with ASD, and 13 matched TD participants (11 males), aged 10-19 years, from local schools. Intellectual abilities were estimated with the Wechsler Intelligence Scale for Children-4th Edition (WISC-IV; Wechsler, 2003) or the Wechsler Adult Intelligence Scale-4th Edition (WAIS-IV; Wechsler, 2008). The two groups did not differ on either age, verbal comprehension index (VCI), perceptual reasoning index (PRI), working memory index, or processing speed index, which were compared using the Mann-Whitney *U* test (Table 1). The VCI and PRI, calculated according to performances on WISC-IV or WAIS-IV subtests, allowed us to ensure that participants had no general impairment of language comprehension or perceptual abilities. However, as expected, the ASD group differed from the TD group on all autism-spectrum quotient scores (AQ; Baron-Cohen et al., 2001) (from *p* = 0.003 to *p* < 0.0001). All participants with ASD were recruited through an autism resource center in Caen (France). Clinicians had previously diagnosed them according to the *Diagnostic and Statistical Manual of Mental Disorders, 5th Edition* (American Psychiatric Association, 2013), confirmed by administering the Autism Diagnostic Interview-Revised (Rutter et al., 2003) and/or the Autism Diagnostic Observation Schedule-Generic (Lord et al., 2000). For all participants, exclusion criteria were attention-deficit disorder with or without hyperactivity, schizophrenia, history of head trauma with loss of consciousness, recent or regular use of alcohol or drugs, chronic neurological or endocrine disorder, medication use likely to interfere with memory measures, and intellectual disabilities assessed with the WISC-IV or WAIS-IV. The study was approved by three committees: an ethics committee, a methodology committee, and the French Data Protection Authority. A relevant ethics committee approved the study (N°. ID RCB: 2014-A00481-46), and all the participants and their parents provided their written consent, in line with committee guidelines.

**Table 1:** Median ages and cognitive data for the ASD and TD groups.

**Age and cognitive data**		**ASD group (*n* = 13) Median (range)**	**TD group (*n* = 13) Median (range)**	***U*-value**	***P*-value**
Age		16.5 (10.4–19.3)	14.8 (12.3–19.2)	66	0.35
IQ	Full scale	98.0 (75.0–128.0)	105.0 (88.0–125.0)	72	0.54
	VCI	111.0 (45.0–146.0)	106.0 (81.0–138.0)	80.5	0.86
	PRI	97.0 (61.0–138.0)	102.0 (71.0–126.0)	75.5	0.66
	WMI	98.5 (70.0–120.0)	97 (58.0–125.0)	83.5	0.98
	PSI	86.5 (72.0–115.0)	100.0 (78.0–115.0)	63.5	0.29
AQ	Total	31.0 (24.0–43.0)	11.0 (4.0–22.0)	0	<0.0001^*^
	Social skills	7.5 (3.0–10.0)	2.0 (0.0–7.0)	7	<0.0001^*^
	Communication skills	6.5 (5.0–9.0)	1.0 (0.0–4.0)	0	<0.0001^*^
	Attention switching	8.0 (4.0–9.0)	2.0 (0.0–5.0)	2.5	<0.0001^*^
	Attention to detail	7.5 (0.0–9.0)	2.0 (0.0–7.0)	26.5	0.003^*^
	Imagination	6.0 (2.0–9.0)	2.0 (0.0–8.0)	27	0.003^*^

*ASD, participants with autism spectrum disorder; TD, typically developing participants; IQ, intelligence quotient; VCI, Verbal Comprehension Index; PRI, Perceptual Reasoning Index; WMI, Working Memory Index; PSI, Processing Speed Index; AQ, autism-spectrum quotient*.

* *In bold: Significant differences were observed between the ASD and TD groups, p < 0.05*.

#### Procedure

In the present study, we used the “From Past to Future Task” already described in a previous publication (Anger et al., 2019), which explores specific past personal events and future thinking for the day before (recent past), last vacation (remote past), next day (near future) and upcoming vacation (distant future). Each participant is asked to produce descriptions of memories or projections with as many details as possible, focusing on the past (i.e., one event that happened yesterday and one last summer vacation; “Can you remember something that happened to you yesterday/last summer vacation? I want you to recall it with plenty of details as if you were reliving this event, and your description has to allow me to imagine this event too”) and the future (i.e., one event that could happen tomorrow and one next summer vacation; “Can you imagine what you might do tomorrow/next summer vacation, either something planned or something completely new, but I want you to imagine what could happen with plenty of details as if you were living this event, and your description has to allow me to imagine this event”). All productions were recorded and manually transcribed (including language mistakes, repeats, and interjections). Only free recall was studied here.

We used an original coding system to explore social identity in autobiographical productions, based on self-categorization theory (Turner and Reynolds, 2011). Free recalls were divided into clauses (one clause per conjugated verb). Each clause was segmented into functional elements, excluding metacognitive items and repeats. A functional element is a set of words articulated around a verbal core that has a particular function in the sentence. We therefore identified the subject + verb (one element), and the circumstantial complements of place, time and manner. All the events were counted, and a score was attributed to each clause according to the number of segments. This coding was intended to allow us to investigate (1) social position in autobiographical narrative (personal, social or neutral references), (2) social context (family or extended circle), and (3) social references in social narratives (self-references, inclusive references, and references to others).

**Figure 1:**
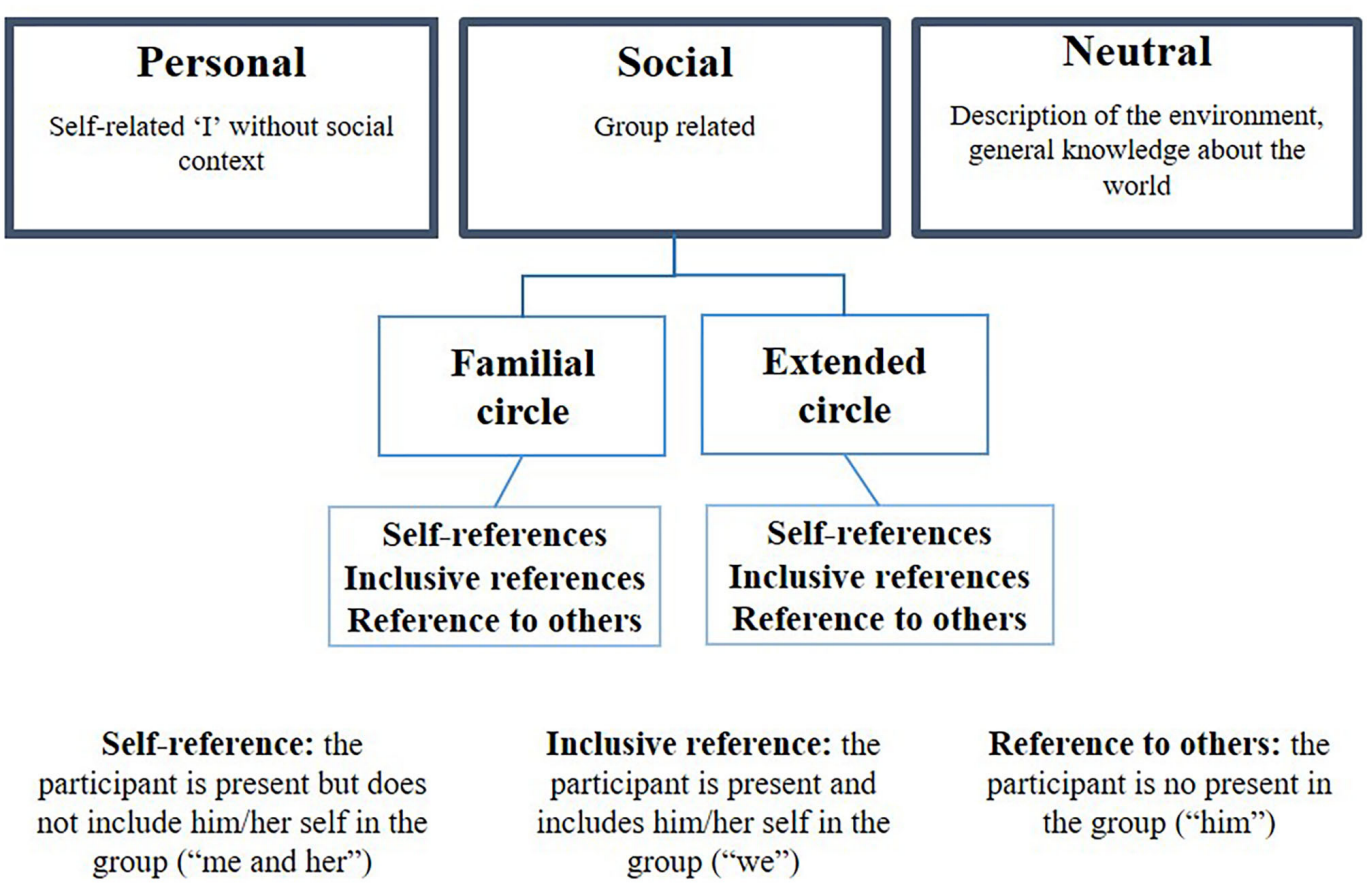
Autobiographical memory coding.

For social position, each clause was thus assigned to one of three categories: *personal* (i.e., relating solely to the participant), *social* (i.e., relating to other people, with or without the participant), or *neutral* (relating to no one). For social context, clauses were assigned to either *family circle* (references to parents, siblings, etc.) or *extended circle* (references to close friends, acquaintances, neighbors, etc.). Finally, for social inclusion in narratives, clauses were assigned to *self-references* (participant present, but not included in the social group; e.g., “me and her”), *inclusive references* (participant present and included in the group; e.g., “we”), or *references to others* (participant not present and not included in the group; e.g., “him”) (Figure 1). After each event, we asked for details about the event, including who was present, in order to assign the correct social category.

For example: “Last night/, I watched/ a movie/ on the couch/ with my cousin” would be scored 5 (five segments or functional elements), and classified as *social* (event related to other people), *family circle* (cousin), and *self-reference* (“I” and “my cousin”). Some sentences might fall into more than one category, in which case the total score would have to be divided between these categories. For example, “Last night/, I watched/ a movie/ on the couch/ with my cousin and my friend” refers both to the family circle (“cousin”) and the extended circle (“friend”). We would therefore divide the score between the two categories [i.e., 2.5 for social, family circle, and self-reference (“I” and “my cousin”), and 2.5 for social, extended circle, and self-reference (“I” and “my friend”)]. All scores were converted into ratios, according to the total number of segments.

All responses were coded by two raters who were blind to participants' diagnoses and the experiment's hypotheses. The raters were provided with short descriptions of each type of scoring category, along with numerous examples of responses that would be placed in each one. Raters were instructed to choose the category they felt best reflected each answer provided by participants. Some answers could be placed in more than one category, on account of say their ambiguity or brevity. Interrater reliability for the repartition into the personal, social and neutral categories was Kappa = 0.93, *p* < 0.001, representing very substantial agreement (Landis and Koch, 1977). Any disagreements were resolved by discussion.

#### Statistical Analyses

Statistical analyses were conducted using Statistica software. Our data did not follow a normal distribution (Shapiro-Wilk, *p* < 0.05), so given the size of our sample, we ran non-parametric analyses. Spearman correlations between age and all scores for each group did not reveal any significant difference, except for neutral clauses in the TD group (*r* = 0.65; *p* = 0.02). We used Mann-Whitney *U* tests to assess between-group differences. Within-group comparisons were calculated using Friedman's test, and *post-hoc* tests with Wilcoxon's test. We calculated Spearman correlations between AQ score (total) and AM scores in the whole group. We also calculated effect sizes (η^2^, Kendall's *W* or *r*, depending on which test was used) adapted for non-parametric data (Fritz et al., 2012).

### Results

All raw scores are presented in Supplementary Table 1. The ASD group produced significantly more segments than the TD group (*U* = 44, *p* = 0.04, η^2^ = 0.17). Between-group analyses of each of the three categories (neutral, personal, and social) did not reveal any significant differences in accuracy (see Table 2). However, we observed a significant difference for the social context focused on the extended circle, where the ASD group produced fewer references than the TD group (*U* = 26, *p* = 0.03, η^2^ = 0.35). Social references also differed from one group to another, as participants with ASD produced significantly fewer self-references (*U* = 25, *p* = 0.002, η^2^ = 0.36) and more references to others (*U* = 43.5, *p* = 0.04, η^2^ = 0.17) than TD. When we considered the two social contexts separately, we observed these performance patterns for references to others in the family circle (*U* = 49, *p* = 0.04, η^2^ = 0.13), and self-references in the extended circle (*U* = 34, *p* = 0.009, η^2^ = 0.26). The ASD group also produced fewer inclusive references associated with the extended circle (*U* = 33, *p* = 0.008, η^2^ = 0.27).

**Table 2:** Median (Range) scores on social references, social context, and social positioning for participants' narratives.

**Category**	**Variables**	**ASD (*n* = 13) Median (range)**	**TD (*n* = 13) Median (range)**	***U*-value**	***p*-value**
	Total number	112 (29–233)	53 (32–161)	44	0.04^*^
Distribution	Neutral	0.17 (0.06–0.39)	0.19 (0–0.33)	77	0.72
	Personal	0.33 (0.02–0.67)	0.34 (0.3–0.62)	79	0.80
	Social	0.52 (0.06–0.84)	0.5 (0.19–0.80)	79.5	0.82
Social context	Family circle	0.29 (0–0.78)	0.1 (0–0.61)	62	0.26
	Extended circle	0.09 (0–0.84)	0.26 (0.10–0.72)	41	0.03^*^
Social references	Self-references	0.03 (0–0.27)	0.13 (0.03–0.37)	25	0.002^*^
	Inclusive references	0.20 (0–0.72)	0.26 (0.06–0.43)	69	0.44
	References to others	0.11 (0–0.37)	0.02 (0–0.25)	43.5	0.04^*^
Social references in family circle	Self-references	0 (0–0.25)	0 (0–0.31)	96	0.64
	Inclusive references	0.12 (0–0.55)	0.05 (0–0.27)	65	0.32
	References to others	0.04 (0–0.27)	0 (0–0.21)	49	0.04^*^
Social references in extended circle	Self-references	0.01 (0–0.11)	0.08 (0–0.20)	34	0.009^*^
	Inclusive references	0.01 (0–0.72)	0.13 (0.03–0.42)	33	0.008^*^
	References to others	0.06 (0–0.19)	0.02 (0–0.22)	48	0.06

*ASD, participants with autism spectrum disorder; TD, typically developing participants*.

* *In bold: Significant differences were observed between the ASD and TD groups, p < 0.05*.

We conducted within-group analyses to compare productions within each category. Friedman analyses revealed significant differences for the distribution of segments, but only in the TD group, χ^2^(2) = 12.52, *p* < 0.001, *W* = 0.54, where participants produced more social references than neutral ones (*z* = 3.18, *p* = 0.001, *r* = 0.89). Concerning social context, participants with ASD produced more references associated with the family circle than with the extended circle (*z* = 2.12, *p* = 0.03, *r* = 0.59). We also observed a different distribution of social references in each group [ASD: χ^2^(2) = 7.84, *p* = 0.02, *W* = 0.30; TD: χ^2^(2) = 13.69, *p* = 0.001, *W* = 0.53]. Participants with ASD produced more self-references and references to others than inclusive references (respectively, *z* = 2.75, *p* = 0.006, *r* = 0.76; *z* = 2.51, *p* = 0.01, *r* = 0.70), whereas TD participants produced more self-references and inclusive references than references to others (respectively, *z* = 2.06, *p* = 0.04, *r* = 0.57; *z* = 3.18, *p* = 0.001, *r* = 0.88). More precisely, and focusing on family circle, Friedman analyses revealed a different distribution for the participants with ASD [χ^2^(2) = 8.97, *p* = 0.01, *W* = 0.34] who produced fewer self-references than either inclusive references or references to others (respectively, *z* = 2.55, *p* = 0.01, *r* = 0.71; *z* = 2.10, *p* = 0.03, *r* = 0.58). Finally, concerning the extended circle, distribution was different for each group [ASD: χ^2^(2) = 9.26, *p* = 0.01, *W* = 0.35; TD: χ^2^(2) = 8.65, *p* = 0.01, *W* = 0.36]. The ASD group made fewer self-references than references to others (*z* = 2.31, *p* = 0.02, *r* = 0.64), and the TD group made more inclusive references than references to others (*z* = 2.98, *p* = 0.003, *r* = 0.83).

We found negative correlations in the whole group between the AQ score (total) and self-references in social circle (*r* = −0.60, *p* < 0.05), self-references in the extended circle (*r* = −0.58, *p* < 0.05), and inclusive references in the extended circle (*r* = −0.40, *p* < 0.05).

### Discussion

In this first study, we investigated social identity through the autobiographical narratives of adolescents with ASD compared with TD, and explored how participants categorized themselves within the different social spheres. First, the distribution of narratives showed a predominance of social references in the TD group, but not in the ASD group. Moreover, participants with ASD associated themselves less with others (self-references) in their narratives, and made more references to others only, compared with TD adolescents. When we considered social circles, the main results revealed that narratives produced by the ASD group focused less on the extended social circle (e.g., friends, acquaintances) than TD narratives did. Their descriptions focused less on inclusive and self-references than TD narratives did. In other words, they produced fewer references in which they associated themselves with someone else when they reported extended circle memories. Furthermore, the decrease of self-references and inclusive references, especially in the extended circle, were associated with high autistic traits.

#### Narrative Distribution

The ASD group produced more autobiographical narratives than the TD group did. Goldman and DeNigris (2015) showed that the amount and length of discourse between children with ASD and their parents about past events did not differ from those of TD families. A meta-analysis highlighted differences in narrative coherence and cohesive adequacy, with ASD participants producing less causally connected narratives than those of a comparison group (Baixauli et al., 2016). Children with ASD incorporate fewer essential elements when freely recalling organized events (McCrory et al., 2007), and produce more disorganized or irrelevant responses (Losh and Capps, 2003).

No significant difference was found between the two groups on the distribution of content (neutral, personal, or social). However, the distribution varied across groups, with more social references (allocentric) in the TD group. Participants with ASD seemed to have no preference for social content in their narratives. This result is consistent with the social difficulties that characterize autism, and with a previous study that reported less socially salient content in the memories of participants with ASD (McCrory et al., 2007).

When we looked as social circle, we found that the ASD narratives focused less on the extended social circle (e.g., friends, acquaintances) than TD narratives did. Within the ASD group, the narratives were more about the family circle than the extended circle, but there was no such difference in the proportions of TD narratives. Social difficulties in ASD make it hard for individuals to interact with people other than family members. Individuals with autism experience difficulties in their social relationships, which are exacerbated during adolescence. This is a period when these relationships are particularly important, and they require far more complex skills than in childhood. People with ASD are fully aware of their difficulties, particularly communication and social interactions, and what they can entail (social rejection, mockery, etc.) (Ke et al., 2018). They generally find it hard making friends and initiating interactions. This has an impact on their autobiographical narratives, which contain fewer references to the extended social circle. A recent study among the adult siblings of individuals with ASD found that aloofness negatively predicted AM specificity (McDonnell and Nuttall, 2018). Aloofness characterizes social disengagement, and may explain the lack of interest in friends or difficulty engaging in socially appropriate interactions in ASD, and thus the paucity of references to extended social circle. Difficulties in social interaction, verbal communication, and socialization disrupt the elaboration and consolidation of these memories (Goddard et al., 2007; Bon et al., 2012).

#### Self-categorization

Concerning social references (self-references, inclusive references, and references to others), the narratives produced by the ASD group contained fewer self-references (egocentric) and more references to others (allocentric) than those produced by the TD group. Regarding the results as a whole, references to others predominated over other positions involving oneself in the ASD group (self-reference and inclusive reference). The opposite pattern was observed among TD participants, as they produced more inclusive references (intracentric). Meta-analysis results also revealed significant differences in the use of referential mechanisms by individuals with ASD, with a greater number of ambiguous pronouns (Baixauli et al., 2016). The predominant use of allocentric discourse was in line with the study by Lind and Bowler (2010), who described a greater “spectator” perspective in this population relative to a comparison group. Millward et al. (2000) also reported better recall of information about others than about self among participants with ASD. Research has also highlighted reduced memory for actions performed by the self than for actions performed by others (Russell and Jarrold, 1999; Russell and Hill, 2001; Robinson et al., 2016). However, recent data are more nuanced. In three experiments, individuals with autism exhibited a self-reference effect similar to that of a comparison group (Lind et al., 2019). Self-reference effect refers to the memory advantage for encoding self-relevant information. These results argue in favor of preserved self-awareness in autism. Our data do not contradict this notion, as we showed that participants with ASD provided a large number of autobiographical productions. However, we go beyond this by showing that the position in social memories may be different from that observed for the TD adolescents, as the ASD group preferred to refer to others when recalling personal memories.

When we focused on social circles, we also observed this predominance of references to others in the ASD group both for the family and the extended circle. The distribution was more balanced in the TD group, with more inclusive references when considering the extended circle. Participants with ASD were less conscious of being themselves in relationships with others (Neisser, 1988; Hobson, 1990; Tomasello, 1995), especially in the extended social circle where ASD participants integrated less themselves with others. They described limited social interaction and provided less relevant information about themselves in relation to these social experiences. Representations of the self with others therefore seem vulnerable. The self appears to be incoherent and hinders the creation of a coherent mental picture of oneself (Cashin, 2005; Bowler et al., 2008, 2011) in line with the weak central coherence theory (Happé and Frith, 2006). Self-awareness appears to be disrupted in ASD (Frith, 1989; Hobson, 1990). The concept of self develops later, and adolescents with ASD generally have a less developed sense of self than TD adolescents (Jamison and Schuttler, 2015). This decrease in self-references may contribute to reduced mental capacity and social functioning, and high autistic traits (Henderson et al., 2009). We also observed that this result was associated with more severe autistic traits. Self-categorization difficulties may also contribute to the atypical specification of AMs and more inefficient use of the self as an effective memory organization principle (Goddard et al., 2017). Beyond that, disruptions in social relations and communication limit the possibilities for social engagement, and thus the opportunities to acquire psychological knowledge about oneself and others (Neisser, 1988; Hobson, 1990). Children with ASD have difficulty incorporating elements of discourse that promote the sharing of memories in a social context, and do not grasp the value of using memory as an object of social interaction (Goldman, 2008).

Overall, our results demonstrate the value of using AM to study social identity in autism, as well as the differences of producing memories about the extended social circle and integrating into a social group. In the light of these results, we would expect working on AM in social skills training to extend social and inclusive references.

## Study 2: AM Rehabilitation

### Introduction

AM events are produced in the context of social relationships, primarily within the family, where communication may be facilitated by shared memories (Goldman and DeNigris, 2015), but also in the proximal social context, which includes people with whom the child is in regular contact. Social interactions within the family or with friends are critical moments where individual memories are shared and where the past becomes a collective construct (Pasupathi, 2001). AM is essential in social interactions, as it supports conversation. It allows social bonds to be developed, maintained, and improved, and collective memories to be shared or co-constructed. AM also plays a role in empathy, improving the understanding of others through the sharing of life events. In turn, this collaborative construction improves sharing and social interactions, and contributes to the development of identity. For these reasons, AM may be an exciting tool for social rehabilitation programs, notably in ASD where social difficulties are at the core of the disorder (American Psychiatric Association, 2013). In their review, Fivush et al. (2006) found that children whose parents engaged in elaborative reminiscing with them (i.e., asking many open-ended, wh- questions such as *when, who, what*, and *where*) later excelled in narration. Similarly, McCabe et al. (2017) described an intervention with the parents of children with ASD about the importance of personal narratives and recommendations for improving narration. Recommendations included talking with the children and asking them direct questions about events. The authors showed that parents in the intervention group provided significantly more elaboration in conversations with their children than parents in the control condition.

To our knowledge, there are currently no AM rehabilitation programs for children or for individuals with ASD. The only ones that have been developed so far concern normal aging (Chiang et al., 2010; Madore and Schacter, 2014), or psychiatric (depression or schizophrenia; Raes et al., 2009; Neshat-Doost et al., 2012; Ricarte et al., 2014; Hitchcock et al., 2017) or neurodegenerative pathologies (Woods et al., 2005, 2012; Lalanne and Piolino, 2013). There are benefits in most cases except for neurodegenerative diseases where results are more controversial and may result from the inevitable cognitive degradation over time. Benefits concern anxiety, communication, social interaction, orientation and general cognitive functioning, as well as sense of identity and personal satisfaction (Haight and Webster, 1995; Cotelli et al., 2012). Interventions have positive effects on AM functioning, with a significant impact on behavior, reducing depression in younger adults (Raes et al., 2009) and enhancing social functioning, notably social problem-solving (Leahy et al., 2018) in older ones. In the present study, we developed a new intervention focusing on social interactions through AM (SIAM). This program aimed to improve conversational skills and, consequently, social interactions and social identity in autism. It featured (1) therapeutic education (Neshat-Doost et al., 2012), (2) induction of episodic specificity by creating an image or a mental video of the memory and remembering a maximum of details (Madore and Schacter, 2014), (3) a recollection procedure based on Conway's model of AM ranging from general personal knowledge to more specific and detailed memories (Piolino, 2008), (4) collective training and assessment of the richness of memories by a peer (Chiang et al., 2010; Ricarte et al., 2014), (5) facilitation and compensation techniques (Lalanne and Piolino, 2013) in line with the task support hypothesis in ASD (Bowler et al., 2004), and (6) personal work (homework, journal) (Neshat-Doost et al., 2012; Ricarte et al., 2014). We also created tools (e.g., use of visual cues and rekindling for impairment in memory retrieval and access; Anger et al., 2019) to remediate other specific difficulties in ASD (**Table 4**, Supplementary Table 2).

The development of this rehabilitation program took the particular features of autism into account. It was administered by a cognitive psychologist, assisted by another medical or paramedical professional. This approach was similar to that used in establishing social skills training groups, whose relevance is now supported by several objective arguments (e.g., Baghdadli et al., 2013). These programs provide explicit training in social skills, based on widely used approaches (emotion, facial recognition, eye contact, tone of voice, etc.) that are highly relevant in ASD. The originality of the present study was that it strengthened AM as an adapted ecological communication tool and improved inclusion and social identity.

### Material and Methods

#### Participants

Two male and one female participant with ASD (Nils, Sam and Allison; names changed to preserve confidentiality) who had been included in Study 1 were randomly selected to participate in the SIAM pilot study, conducted at the autism resource center. This intervention was carried out in the context of ongoing social skills programs within the center.

Three participants with autism from Study 1 (Arthur, Steeve and Will; names changed to preserve confidentiality) who were not included in the rehabilitation program, were randomly selected for a comparison follow-up. They were retested a year later as part of their regular follow-up. Table 3 summarizes participants ages and cognitive data.

**Table 3:** Mean ages and cognitive data for the three ASD participants included in SIAM program (Allison, Nils and Sam) and for three ASD participants controls (Arthur, Steeve and Will).

**Ages and cognitive data**		**Allison**	**Nils**	**Sam**	**Arthur**	**Steeve**	**Will**
Age		16.3	12.9	16.3	16.6	12.7	18.1
QI	Full scale	96	108	75	78	97	120
	VCI	108	130	114	84	96	120
	PRI	96	92	61	72	94	138
	WMI	103	85	70	94	109	120
	PSI	78	106	76	78	93	72
AQ	Total	27	24	30	28	32	34
	Social skills	8	3	10	6	9	8
	Communication skills	7	5	7	6	5	5
	Attention switching	9	6	9	6	7	8
	Attention to detail	1	8	0	4	6	7
	Imagination	2	2	4	6	5	6

*IQ, intelligence quotient; VCI, Verbal Comprehension Index; PRI, Perceptual Reasoning Index; WMI, Working Memory Index; PSI, Processing Speed Index; AQ, autism-spectrum quotient*.

#### Intervention

**Figure 2:**
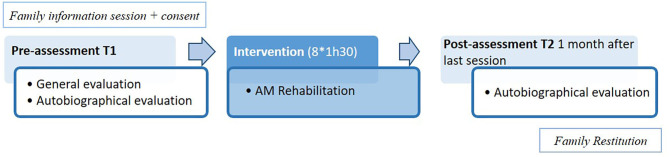
General procedure of the SIAM rehabilitation program.

The experimental SIAM intervention consisted of 8 weekly 90-min sessions. Participants were included for 4 months, with 8 weeks of the intervention program (and 2 additional weeks for vacations). They underwent pre- (T1) and post-assessments (T2) of AM, together with a general cognitive evaluation. The pre-assessment was in fact the Study 1 assessment. Before the beginning of the sessions, we met each family to present the program and answered all their questions. The last appointment allowed a debriefing. Figure 2 shows the general organization of the intervention.

The program was rolled out by therapists who had training and expertise in working with children with ASD. It comprised eight individual and group sessions. Individual sessions featured personalized exercises, while group sessions allowed personal memories and knowledge to be shared, with a view to improving social communication. The 30-min individual sessions were conducted with a therapeutic education approach, and include preparation for the next collective session. They gave participants an opportunity to reformulate the previous group session and focus on their difficulties. Each child was followed by the same therapist throughout the intervention. Each group session started with a “daily mood” supported by photos and names of emotions, after which participants were given the theme of the day's session. Group sessions were designed to improve participants' narrative and conversational skills, by encouraging them to share personal memories and knowledge, thus creating common social interaction bonds. At the end of each session, the therapists took stock of the content discussed during the session. Participants were given assignments for the next session (e.g., bringing an object and explaining how they came to have it, writing down a memory, describing memories to the family during the week, etc.). The contents of each session are summarized in Table 4 (see also Supplementary Table 2 for detailed sessions). A workbook was created for each of the participants, so that they would have a written record. It included an overview of the sessions, a timeline of the sessions, a summary of important points mentioned in each session, tools addressed (AM map, key phrases, time representation, schedule, etc.).

**Table 4:** Content of each session.

**Session**	**Individual sessions (30 min)**	**Collective sessions (1 h)**
1	Therapeutic education on AM functioning (semantic component)	Group rules and objectives Getting to know each other (sharing personal knowledge)
2	Therapeutic education on AM functioning (episodic component)	Familiarization with memory elements (what, where, who…)
3	Reminder of terms Creating a mental map of AM	Adapting the content of memories to the interlocutor (roleplay)
4	Time perception and projection	Organizing and planning a shared event (snack) together
5	Sharing an event (semistructured): encoding	
6	General feedback on the snack (emotion, perceptions, continuity, etc.)	Recall and confrontation of different points of view about the semistructured event
7	Memory and temporality Recollection cues	Sharing old memories
8	Assessment of the sessions and the material produced (memory box, etc.)	
	Identification of the other based on description	
	Assessment of knowledge about AM (game)	

#### Statistical Analyses

We compared each participant's scores with those of the group in Study 1, and calculated *z*-scores. As the comparison group consisted of 13 TD participants, we set the pathological threshold at 2.179 (*p* < 0.05, two-sided). We also compared the changes between T1 and T2 with a modified *z*-score derived from Mellenbergh and van den Brink (1999), calculated according to the following formula: (T1–T2)/standard deviation of the comparison group.

### Results

**Figure 3:**
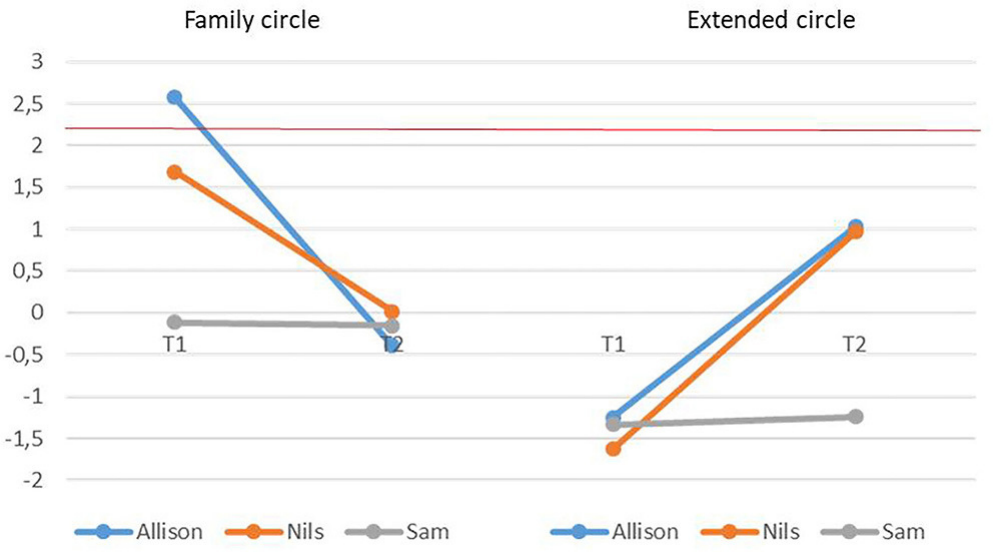
Narratives associated with family circle (left) and extended circle (right) produced during the first (T1) and second (T2) assessments. The red line represents the pathological cut-off score (*z* = 2.179).

**Figure 4:**
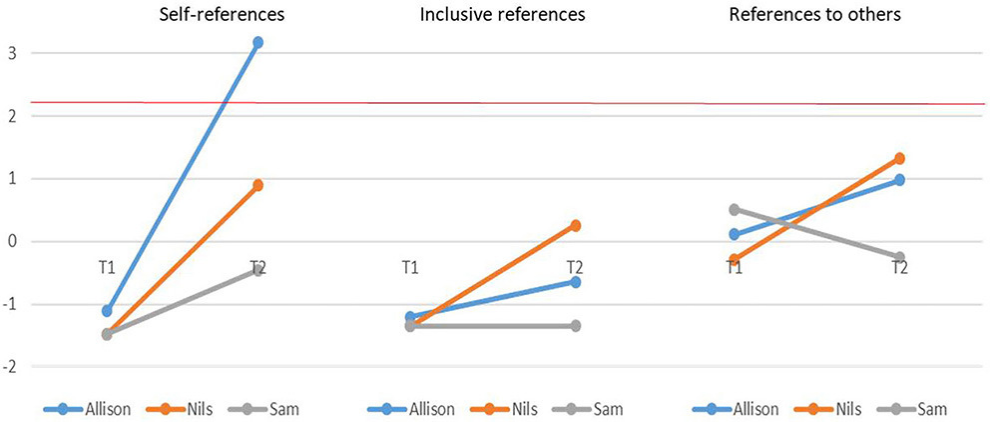
Social references associated with the extended circle: self-references (left), inclusive references (center), and references to others (right) during the first (T1) and second (T2) assessments. The red line represents the pathological cut-off score (*z* = 2.179).

Results showed that Nils produced significantly more narratives than the comparison group at T1 (Table 5). The same was true for Sam at T2. Allison produced more social references concerning the family circle than the comparison group at T1 (Figure 3). She also produced more references to others at T1, and trended to report more self-references at T2 (*z* = 2.167). Regarding narratives associated with the family circle, two participants (Allison and Nils) produced more references to others at T1, and these scores normalized at T2. Finally, Allison had a high self-reference score for extended circle at T2 (Figure 4). The other participants' scores also increased, but remained within the normal range.

**Table 5:** Social reference, social context and social positioning scores for the three ASD participants included in SIAM program (Allison, Nils and Sam) and for three ASD participants controls (Arthur, Steeve and Will).

**Category**	**Variables**	**Allison**		**Nils**		**Sam**		**Arthur**		**Steeve**		**Will**		**TD (*n* = 13) Median (range)**
		**T1**	**T2**	**T1**	**T2**	**T1**	**T2**	**T1**	**T2**	**T1**	**T2**	**T1**	**T2**	
	Total number	85	102	210^*^	144	55	167^*^	29 47	47	60	35	112	124	53 (32–161)
Distribution	Neutral	0.06	0.11	0.07	0.19	0.38	0.13	0.17	0.40^*^	0.07	0.37	0.15	0.23	0.19 (0–0.33)
	Personal	0.15	0.31	0.40	0.15	0.40	0.65	0.21	0.13	0.15	0.03	0.31	0.44	0.34 (0.3–0.62)
	Social	0.79	0.57	0.54	0.65	0.22	0.23	0.62	0.47	0.78	0.6	0.54	0.33	0.5 (0.19–0.80)
Social context	Family circle	0.70^*^	0.09	0.52	0.17	0.15	0.14	0.41	0.47	0.78^*^	0.34	0.29	0.26	0.1 (0–0.61)
	Extended circle	0.09	0.49	0.02	0.48	0.07	0.09	0.21	0.00	0.00	0.26	0.24	0.07	0.26 (0.10–0.72)
Social references	Self-reference	0.27	0.36	0.00	0.18	0.02	0.10	0.07	0.06	0.00	0.00	0.04	0.04	0.13 (0.03–0.37)
	Inclusive reference	0.26	0.12	0.31	0.35	0.09	0.08	0.55^*^	0.30	0.55^*^	0.37	0.20	0.26	0.26 (0.06–0.43)
	Reference to others	0.26^*^	0.10	0.23	0.12	0.11	0.05	0.00	0.11	0.23	0.23	0.30^*^	0.03	0.02 (0–0.25)
Social references in Family circle	Self-reference	0.25	0.06	0.00	0.03	0.02	0.03	0.00	0.06	0.00	0.00	0.00	0.00	0 (0–0.31)
	Inclusive reference	0.24	0.03	0.31	0.14	0.09	0.08	0.41^*^	0.30	0.55^*^	0.29	0.13	0.26	0.05 (0–0.27)
	Reference to others	0.21^*^	0.00	0.21^*^	0.00	0.04	0.02	0.00	0.11	0.23^*^	0.06	0.17^*^	0.00	0 (0–0.21)
Social references in Extended circle	Self-reference	0.02	0.30^*^	0.00	0.15	0.00	0.07	0.07	0.00	0.00	0.00	0.04	0.04	0.08 (0–0.20)
	Inclusive reference	0.02	0.09	0.00	0.20	0.00	0.00	0.14	0.00	0.00	0.09	0.07	0.00	0.13 (0.03–0.42)
	Reference to others	0.05	0.10	0.02	0.12	0.07	0.02	0.00	0.00	0.00	0.17	0.13	0.03	0.02 (0–0.22)

TD, typically developing participants;

* *In bold: p < 0.05 (−2.179 > z and z > 2.179), p-values refer to modified z score with standard deviation of the comparison group*.

Within-participants comparisons revealed an increase in the total number of productions between T1 and T2 for Sam (*z* = −2.89, *p* = 0.05), accompanied by a decrease in the proportion of neutral ones (*z* = 2.69, *p* = 0.05). We observed a decrease in social references concerning the family (*z* = 2.97, *p* = 0.05), but an increase in references concerning the extended circle for both Allison (*z* = −2.27, *p* = 0.05) and Nils (*z* = −2.59, *p* = 0.05). There were significant decreases in references to others in the family circle for both Allison (*z* = 3.51, *p* = 0.01) and Nils (*z* = 3.45, *p* = 0.01). We observed more moderate decreases in self-references (*z* = 2.03) and inclusive references (*z* = 2.01) for Allison. For these two participants, self-references associated with the extended circle significantly increased between T1 and T2 (Allison: *z* = −4.28, *p* = 0.01; Nils: *z* = −2.37, *p* = 0.05).

Statistical analyses on controls revealed that at T1, Arthur and Steeve produced significantly more inclusive references than the comparison group, especially in family circle (Table 5). Will and Steeve provided more references to others in family circle, extended to the whole social circle only for Will. In addition, Steeve had a high family circle narrative score. All scores at T2 were similar to the comparison group except for an increase in neutral proportion of narratives for Arthur.

Within comparisons between T1 and T2 showed an increase in the number of neutral narratives for Arthur and Steeve (*z* = −2.43; *z* = −3.20, *p* = 0.05). A decrease in reference to others was observed for Will concerning the whole social domain (*z* = 3.19, *p* = 0.05) and the family circle (*z* = 2.81, *p* = 0.05). Steeve also showed a decrease in reference to others (*z* = 2.92, *p* = 0.05) and inclusive references (*z* = 2.51, *p* = 0.05) associated with family circle, between T1 and T2.

#### Qualitative Observations

Clinical observations showed that all three adolescents improved their social recall of memories, reporting more detailed and appropriate events. Social interactions were facilitated: they spontaneously asked for more details about a memory recounted by a peer, one of them increasingly took the initiative in interactions, not hesitating to help the others or bring them back into the discussion to obtain more information. They enjoyed sharing memories together, and the families confirmed this impression. Both the adolescents and their parents expressed positive opinions during the family debriefing meeting. The therapists observed active cooperation and engagement of teenagers in each session.

### Discussion

Three participants participated in an original rehabilitation program focusing on AM. Preliminary results showed that narratives produced during pre- and post-rehabilitation assessments were distributed in the same way as in the TD group. However, other scores changed. One of the adolescents with ASD provided more social references to the family in T1, that normalized after rehabilitation and the two others increased their references to the extended circle. These two adolescents provided more self-references concerning the extended circle after rehabilitation. This may have reflected a social openness that went beyond the family framework, with productions including more people. We also showed with three ASD participants who did not follow our intervention, limited changes after 1 year concerning the narratives on the social spheres.

These initial results appear to support the relevance of this approach. More broadly, we noted the value of the referring therapists, and both the individual and group sessions. The individual sessions allowed for personalized work and provided an opportunity to discuss with the therapist, while the group sessions focused on sharing memories that reinforced social interactions. The sharing of memories in a small ASD group could be the key to taking charge of social identity through AM.

The social communication difficulties observed in individuals with ASD may result from a disability to interact with peers and/or a lack of desire to do so, with the social criterion being seen as less important (Russell and Jarrold, 1999; Lind and Bowler, 2009). These disabilities may be consistent with insufficient appreciation of the basic communication rules, such as exchanges of autobiographical events. AM is consolidated through the act of storytelling in social relationships, and is regulated by communication rules. It is constructed from experiences of events, but is consolidated and reshaped through interactions with others. AM facilitates social engagement through shared memories, and is enhanced by sharing narrative construction and exchanging memories with others (Nelson and Fivush, 2004). We can assume that working on AM has repercussions on personal and social identity by reinforcing the continuity of individuals' identity over time, and thus the self's cohesion (Ricarte et al., 2014).

These observations support our hypotheses concerning the benefits of collective involvement and the importance of shared memory mechanisms. They lead us to propose adjustments to current therapies, particularly concerning parental involvement, in order to foster exchanges within the family first, then with an extended social circle to help construct this shared memory (McCabe et al., 2017). Production of AMs occurs in social relationships, primarily within the family, where social relationships are strong and many shared memories are formed (Goldman and DeNigris, 2015). The parents of children with ASD use specific strategies (e.g., more direct questions, more corrections) to trigger exchanges with their children, in order to overcome the difficulties they encounter and improve their children's participation (Goldman and DeNigris, 2015). Compared with social skills training groups, the present project's originality lay in the fact that it relied on personalized and ecological material, namely AMs. This approach allowed participants to gain a better understanding of their personal identity and relations with others through the sharing of memories, and by so doing helped them to develop and reinforce their social identity and integration.

Although these results are only preliminary, they are also encouraging. They confirm the value of memory as a vector of social interactions. The present study also highlighted the usefulness of combining several methods, such as individual and collective sessions, therapeutic education, roleplay, etc. It is now important to extend this program to other children and to evaluate the specificity of the intervention. The next step following this pilot study will be to compare its results with those obtained with other therapies such as social skills training. This has a broader social objective, as groups contain larger numbers of participants, and there are no individual and personalized sessions. However, they are led by one or more therapists and include roleplay and numerous verbal interactions.

## General Discussion

AM enables the construction and maintenance of a sense of identity and continuity over time (Tulving, 1985). It is key to social interactions, enabling the development and maintenance of social ties, and contributes to the sharing and co-construction of collective memory. AM in autism is more fragmented, making access to personal events more complicated, with a decrease in phenomenological richness associated with limited social skills (Losh and Capps, 2003; Goddard et al., 2014). These difficulties have repercussions on memory consolidation. Even if AM does not lie at the core of ASD, it appears to be a relevant object of study, and can be used to assess other skills such as identity and social integration. AM is a relevant and ecological target for rehabilitation, allowing therapists to work not just on the functioning of AM itself, but also more broadly on wider functions such as social, directive, and identity functions.

Our two studies yielded new findings on social positioning in the autobiographical narratives of individuals with ASD, the value of AM as a vector of social interaction, and how it can be used in a rehabilitation program. We showed that ASD narratives focus more on the family than on extended social spheres, compared with TD narratives. ASD participants included themselves less in the social group in their descriptions and produced more references to others than TD adolescents did; in others words, adolescents with ASD were less egocentric and more allocentric. This tendency to focus narratives on the family can be regarded as a peculiarity of autism that does not necessarily require remediation if individuals do not express a specific complaint. Our rehabilitation program was not intended to directly encourage participants to interact with people outside their family circle. However, post-test AM results showed that promoting interactions using AM did indeed extend the social circle to include non-family members. In a natural way, the program increased extra-family narrative references by the two youngest adolescents, who produced more social integration markers. We also qualitatively noted more frequent initiative-taking in interactions. At a more “human” level, participants and their parents reported a sense of well-being during the sessions, with genuine motivation and compliance. The participants particularly appreciated the opportunity to talk and exchange in a small group.

Impaired social references in autobiographical narratives can impact the self-awareness and psychological self-representation of individuals with ASD (Frith, 1989; Hobson, 1990; Lee et al., 1994; Millward et al., 2000; Lind, 2010). They may contribute to difficulty recalling social memories related to information about the self (Millward et al., 2000; Henderson et al., 2009; McDonnell et al., 2017) or to a limited vocabulary for describing internal states (Bang et al., 2013; Lind et al., 2014). These modifications may make the processes that organize information in memory less effective (Goddard et al., 2017), thus impacting self-understanding and awareness of one's own experiences, and ultimately contributing to the self's dissociation from AM.

Events we experience are integrated and organized around internal information. They are adjusted according to our previous experiences and involve the working self (Conway and Pleydell-Pearce, 2000). Memory recollection with a feeling of reliving emerges from the coherence of the present self with the past self, making it possible to relive perceptual-sensory details (Baker-Ward et al., 1997; Ornstein et al., 1998). Thus, impaired narrative references to the social context hinder the retrieval of autobiographical events and may explain the difficulties found in autism.

Other studies report memories with less socially salient content (McCrory et al., 2007) and fewer elements associated with personal meaning (Goldman, 2008). Adolescents with ASD appear to compensate for these difficulties in AM for events by preferentially relying on their semantic knowledge. Providing visual aids or giving them more time allows them to adjust their retrieval and reconstruct a more adequate representation of memory (Bruck et al., 2007; Goldman, 2008; Crane et al., 2010; Bon et al., 2012; Brown et al., 2012; Terrett et al., 2013; Goddard et al., 2014; Lind et al., 2014; Anger et al., 2019). We designed our rehabilitation program accordingly.

These difficulties related to the self presumably contribute to decreased mentalization and social functioning capacity, especially concerning people outside the family circle. These disruptions in access to information in long-term memory could also be related to executive dysfunction (Boucher, 2007). AM is correlated with executive functions in children with ASD (Dalgleish et al., 2007; McCrory et al., 2007; Maister et al., 2013; Goddard et al., 2014), and more specifically with the ability to integrate and organize information in memory, mental flexibility, and verbal fluency (Maister et al., 2013; Goddard et al., 2014). We highlighted that decreased egocentric and intracentric references were related to high autistic traits, confirming Ciaramelli et al. (2018) results. They found that AM difficulties are also associated to the severity of ASD symptoms and ToM deficits. Impaired ToM, recognition of one's psychological states, and self-understanding, may all impact the narration of episodic events (Losh and Capps, 2003; Goldman, 2008; McCabe et al., 2013; Kristen et al., 2014). Indeed, these two skills shared both cerebral (Spreng et al., 2009) and cognitive similarities (Buckner and Carroll, 2007; Hassabis and Maguire, 2007). Further studies are needed to interpret this atypical functioning in an integrative way, in relation to the different cognitive theories of autism, such as defects in ToM, executive dysfunction, and weak central coherence.

We report promising results, but given the very limited sample size, they need to be replicated with a larger cohort before they can be generalized. The rehabilitation program also needs to be tested among other adolescents, with a comparison group receiving another form of rehabilitation involving a therapist (usual care or, if possible, social skills training). This would allow us to evaluate more precisely the added value of using AM to enhance social integration. Finally, linking these profiles to an exploration of other cognitive functions would also be relevant, as it would shed light on AM functioning and, more broadly, on social integration difficulties in autism.

## Data Availability Statement

The raw data supporting the conclusions of this article will be made available by the authors, without undue reservation.

## Ethics Statement

The studies involving human participants were reviewed and approved by French ethic committe N° ID RCB: 2014-A00481-46. Written informed consent to participate in this study was provided by the participants' legal guardian/next of kin.

## Author Contributions

PW and BG-G designed the study. JM, AB, EZ, and PW collected the data. AB and PW participated to the cotation. BG-G performed the statistical analyses. PW and BG-G wrote the first complete draft of the manuscript. J-MB, FG, and FE provided substantial modification to the manuscript. All authors read and approved the final manuscript.

## Conflict of Interest

The authors declare that the research was conducted in the absence of any commercial or financial relationships that could be construed as a potential conflict of interest. The reviewer AA declared a past collaboration with one of the authors FE to the handling editor.
