# Supplementary material for: Autobiographical Memory and Social Identity in Autism: Preliminary Results of Social Positioning and Cognitive Intervention
Source: Front Psychol. 2021 Mar 17;12:641765. doi: 10.3389/fpsyg.2021.641765 (PMC8009988; doi:10.3389/fpsyg.2021.641765)
Supplement: Supplementary file 1 [file Table_1.DOCX]

**Supplementary data**

[Table 1. Individual score on social references, social context and social position for participants’ narratives 2](#_Toc63237394)

[Table 2: Details of sessions. All collective sessions started with “daily mood” and finished with highlights of the session 3](#_Toc63237395)

# Table 1. Individual score on social references, social context and social position for participants’ narratives

| **Participants** | Group | Total number | **Repartition** | | | **Social context** | | **Social references** | | | **Social references**  **in Family circle** | | | **Social references**  **in Extended circle** | | |
| --- | --- | --- | --- | --- | --- | --- | --- | --- | --- | --- | --- | --- | --- | --- | --- | --- |
|  |  |  | Neutral | Personal | Social | Family circle | Extended circle | Self-reference | Inclusive reference | Reference to others | Self-reference | Inclusive reference | Reference to others | Self-reference | Inclusive reference | Reference to others |
| A01 | ASD | 161 | 0,14 | 0,02 | 0,84 | 0,00 | 0,84 | 0,03 | 0,72 | 0,09 | 0,00 | 0,00 | 0,00 | 0,03 | 0,72 | 0,09 |
| A02 | ASD | 60 | 0,07 | 0,15 | 0,78 | 0,78 | 0,00 | 0,00 | 0,55 | 0,23 | 0,00 | 0,55 | 0,23 | 0,00 | 0,00 | 0,00 |
| A03 | ASD | 49 | 0,27 | 0,67 | 0,06 | 0,00 | 0,06 | 0,00 | 0,00 | 0,06 | 0,00 | 0,00 | 0,00 | 0,00 | 0,00 | 0,06 |
| A04 | ASD | 233 | 0,33 | 0,15 | 0,52 | 0,39 | 0,12 | 0,05 | 0,09 | 0,38 | 0,05 | 0,07 | 0,27 | 0,00 | 0,02 | 0,11 |
| A05 | ASD | 114 | 0,39 | 0,33 | 0,27 | 0,00 | 0,27 | 0,06 | 0,15 | 0,06 | 0,00 | 0,00 | 0,00 | 0,06 | 0,15 | 0,06 |
| A06 | ASD | 131 | 0,27 | 0,67 | 0,06 | 0,00 | 0,06 | 0,01 | 0,02 | 0,04 | 0,00 | 0,00 | 0,00 | 0,01 | 0,02 | 0,04 |
| A07 | ASD | 112 | 0,15 | 0,31 | 0,54 | 0,29 | 0,24 | 0,04 | 0,20 | 0,30 | 0,00 | 0,13 | 0,17 | 0,04 | 0,07 | 0,13 |
| A08 | ASD | 168 | 0,33 | 0,37 | 0,30 | 0,29 | 0,01 | 0,02 | 0,23 | 0,05 | 0,02 | 0,23 | 0,04 | 0,00 | 0,00 | 0,01 |
| A09 | ASD | 29 | 0,17 | 0,21 | 0,62 | 0,41 | 0,21 | 0,07 | 0,55 | 0,00 | 0,00 | 0,41 | 0,00 | 0,07 | 0,14 | 0,00 |
| A10 | ASD | 94 | 0,10 | 0,40 | 0,51 | 0,21 | 0,30 | 0,13 | 0,13 | 0,24 | 0,02 | 0,13 | 0,06 | 0,11 | 0,00 | 0,19 |
| A11 | ASD | 85 | 0,06 | 0,15 | 0,79 | 0,70 | 0,09 | 0,27 | 0,26 | 0,26 | 0,25 | 0,24 | 0,21 | 0,02 | 0,02 | 0,05 |
| A12 | ASD | 210 | 0,07 | 0,40 | 0,54 | 0,52 | 0,02 | 0,00 | 0,31 | 0,23 | 0,00 | 0,31 | 0,21 | 0,00 | 0,00 | 0,02 |
| A13 | ASD | 55 | 0,38 | 0,40 | 0,22 | 0,15 | 0,07 | 0,02 | 0,09 | 0,11 | 0,02 | 0,09 | 0,04 | 0,00 | 0,00 | 0,07 |
| C01 | TD | 33 | 0,15 | 0,59 | 0,26 | 0,00 | 0,26 | 0,20 | 0,06 | 0,00 | 0,00 | 0,00 | 0,00 | 0,20 | 0,06 | 0,00 |
| C02 | TD | 37 | 0,19 | 0,62 | 0,19 | 0,00 | 0,19 | 0,08 | 0,11 | 0,00 | 0,00 | 0,00 | 0,00 | 0,08 | 0,11 | 0,00 |
| C03 | TD | 53 | 0,30 | 0,25 | 0,45 | 0,19 | 0,26 | 0,13 | 0,32 | 0,00 | 0,00 | 0,19 | 0,00 | 0,13 | 0,13 | 0,00 |
| C04 | TD | 65 | 0,17 | 0,03 | 0,80 | 0,61 | 0,19 | 0,13 | 0,43 | 0,25 | 0,13 | 0,27 | 0,22 | 0,00 | 0,16 | 0,03 |
| C05 | TD | 32 | 0,19 | 0,34 | 0,47 | 0,23 | 0,23 | 0,28 | 0,19 | 0,00 | 0,14 | 0,09 | 0,00 | 0,14 | 0,09 | 0,00 |
| C06 | TD | 101 | 0,06 | 0,44 | 0,50 | 0,34 | 0,17 | 0,17 | 0,24 | 0,10 | 0,07 | 0,21 | 0,06 | 0,10 | 0,03 | 0,04 |
| C07 | TD | 161 | 0,16 | 0,34 | 0,51 | 0,00 | 0,51 | 0,13 | 0,35 | 0,02 | 0,00 | 0,00 | 0,00 | 0,13 | 0,35 | 0,02 |
| C08 | TD | 48 | 0,00 | 0,42 | 0,58 | 0,48 | 0,10 | 0,38 | 0,21 | 0,00 | 0,31 | 0,17 | 0,00 | 0,06 | 0,04 | 0,00 |
| C09 | TD | 36 | 0,33 | 0,28 | 0,39 | 0,10 | 0,29 | 0,06 | 0,26 | 0,07 | 0,06 | 0,05 | 0,00 | 0,00 | 0,21 | 0,07 |
| C10 | TD | 36 | 0,14 | 0,14 | 0,72 | 0,00 | 0,72 | 0,08 | 0,42 | 0,22 | 0,00 | 0,00 | 0,00 | 0,08 | 0,42 | 0,22 |
| C11 | TD | 99 | 0,22 | 0,23 | 0,55 | 0,00 | 0,55 | 0,20 | 0,34 | 0,01 | 0,00 | 0,00 | 0,00 | 0,20 | 0,34 | 0,01 |
| C12 | TD | 74 | 0,24 | 0,48 | 0,28 | 0,00 | 0,28 | 0,08 | 0,12 | 0,08 | 0,00 | 0,00 | 0,00 | 0,08 | 0,12 | 0,08 |
| C13 | TD | 101 | 0,31 | 0,19 | 0,50 | 0,25 | 0,26 | 0,03 | 0,37 | 0,11 | 0,00 | 0,23 | 0,01 | 0,03 | 0,13 | 0,09 |

# Table 2: Detailed Content of each session.

All collective sessions started with daily mood and finished with highlights of the session

| **Session** | **Individual sessions (30 min)** | **Collective sessions (1hr)** | | **Mission** |
| --- | --- | --- | --- | --- |
| **1** | - Explanation of how sessions are conducted - Brief presentation of each session - Therapeutic Education on AM functioning (Semantic component)   - Self-Knowledge: character, family, beliefs, etc.   - Utility: social, identity, directive functions | - Presentation: give two personal details - Group rules and objectives - Summary of the individual session - Getting to know each other (Sharing personal knowledge)   - Game inspired by “snakes and ladders” with a personal information to detail (favorite movie, siblings, a quality, a defect, etc.) | | Bring back an object and tell how you got it |
| **2** | - Reminder of the previous session - Therapeutic education on AM functioning (Episodic component)   - Specific events (what, where, when, who, how) in past and future   - Processings: storage, retrieval, repetition, motivation, self-relevance, etc.   - How to mention it in a conversation? | - Summary of the individual session - Familiarization with memory elements using a grid (what, where, who…)   - Mission: tell a memory related to the personal object, other participants are asked if there are any missing elements - Feedback: difficulty, why it's important? What does it bring to others? | | Writing a memory using the grid |
| **3** | - Reminder and synthesis of the previous sessions - Creating a mental map of AM (Self-Knowledge, specific personal events, utility) | - Mission: Read the memory, participants ask questions (grid) + free questions - Adapting the content of memories to the interlocutor (to whom, how, goal; roleplay game) | | Telling a memory of the day to parents during the week |
| **4** | - Reminder of the previous session - Mission feedback - Time perception and projection into the future   - Based on our past experience and knowledge   - Utility: plan, project, anticipate - Role-playing (with visual cues): organize a snack: what, who, steps, etc. | - Organizing and planning a shared event (snack) together - Exchanging of ideas about each other's snack experiences - Imagining how the snack could take place by picking the different elements (who, how, perception...) - Planning: Who brings what back? What game? | | Bring a snack, a drink or cakes |
| **5** | - Sharing an event (semi-structured) together: encoding | | |  |
|  | - 1. Task distribution, organization: setting up of the snack table   2. Game around the different sensations (odor lottery)   3. Significant event: interruption of an unknown person | | - 1. Snack   2. Game on the representation of time   3. Group photo |  |
| **6** | - General feedback on the snack (how it happened, appreciation, talk to those around you, etc.) | - Recall and confrontation of different points of view about the snack - Feelings, interrogations, self-appreciation, self and other references, the pleasure of remembering and recounting a fun event. | | Tell the snack to two people |
| **7** | - Memory and temporality   - Place on a timeline the events recounted by the adolescent during the sessions. - Tips to remember old events   - Gateway: where, what, who, when, how   - Cues: photo, movie, ask someone, journal | - Mission feedback - Sharing old memories   - Game inspired by snakes and ladders with “the first time” (the first time I made a friend, I was afraid, I went to a movie, etc.) | | Complete a silhouette with drawings of memories that make your identity. |
| **8** | - Assessment of the sessions and the material produced (memories box, etc.) - Mission feedback: Identification of the other based on their description - Assessment of knowledge about AM (game) | | |  |
